# Morphological and genetic factors shape the microbiome of a seabird species (*Oceanodroma leucorhoa*) more than environmental and social factors

**DOI:** 10.1186/s40168-017-0365-4

**Published:** 2017-10-30

**Authors:** Douglas S. Pearce, Brian A. Hoover, Sarah Jennings, Gabrielle A. Nevitt, Kathryn M. Docherty

**Affiliations:** 1 Department of Biological Sciences, Western Michigan University, 1903 W Michigan Ave, Kalamazoo, MI 49008 USA; 2 Department of Neurobiology, Physiology, and Behavior, College of Biological Sciences, One Shields Avenue, University of California, Davis, CA 95616 USA

**Keywords:** Leach’s storm petrel, Skin microbiome, Brood patch, Uropygial gland

## Abstract

**Background:**

The microbiome provides multiple benefits to animal hosts that can profoundly impact health and behavior. Microbiomes are well-characterized in humans and other animals in controlled settings, yet assessments of wild bird microbial communities remain vastly understudied. This is particularly true for pelagic seabirds with unique life histories that differ from terrestrial bird species. This study was designed to examine how morphological, genetic, environmental, and social factors affect the microbiome of a burrow-nesting seabird species, Leach’s storm petrel (*Oceanodroma leucorhoa*). These seabirds are highly olfactory and may rely on microbiome-mediated odor cues during mate selection. Composition and structure of bacterial communities associated with the uropygial gland and brood patch were assessed using 16S rRNA amplicon-based Illumina Mi-Seq analysis and compared to burrow-associated bacterial communities. This is the first study to examine microbial diversity associated with multiple body sites on a seabird species.

**Results:**

Results indicate that sex and skin site contribute most to bacterial community variation in Leach’s storm petrels and that major histocompatibility complex (MHC) genotype may impact the composition of bacterial assemblages in males. In contrast to terrestrial birds and other animals, environmental and social interactions do not significantly influence storm petrel-associated bacterial assemblages. Thus, individual morphological and genetic influences outweighed environmental and social factors on microbiome composition.

**Conclusions:**

Contrary to observations of terrestrial birds, microbiomes of Leach’s storm petrels vary most by the sex of the bird and by the body site sampled, rather than environmental surroundings or social behavior.

**Electronic supplementary material:**

The online version of this article (10.1186/s40168-017-0365-4) contains supplementary material, which is available to authorized users.

## Background

Microbiomes powerfully impact animal health and behavior [1, 2]. These symbiotic microbial networks have been primarily characterized in humans and other animals in controlled settings, but the relationship between microorganisms and wild animals, particularly in non-mammalian species, remains vastly understudied [3]. Understanding this “second genome” of wild animals is a critical step toward unraveling host-microbe co-evolutionary relationships [4], developmental and genomic interactions [5], and animal behaviors, including mating, predation, and self-recognition [6–8]. Better understanding of these processes using microbially informed approaches will aid in wildlife management, pathogen prevention, and wildlife veterinary practices. To date, the majority of wild animal microbiome studies focus on terrestrial mammals [7–9] and, comparatively, little is known about avian species [10], which account for over 15% of all vertebrates [11]. Most avian microbiome studies have focused on terrestrial birds, which are comparatively more accessible than seabirds. However, the lessons learned from these studies may not apply to seabirds, which have many distinctive life history characteristics. Most obviously, all seabird species spend the majority of their lives at sea and in flight, returning to land only to breed, so their interactions with environmental microorganisms differs drastically from terrestrial birds that may be in constant contact with soil, vegetation, and other birds. Additionally, seabirds provide important nutrient inputs to island and coastal habitats [12] and may serve as important vectors for microbial biogeographical distribution along their vast migration pathways. However, it is unknown what factors contribute to seabird microbiomes and the microbial communities they disperse.

Previous microbiome research suggests that physical, historical, genetic, environmental, and social factors may all influence a host animal microbiome. For example, microbial colonization on human skin depends more on the topographical location than on the age, race, or sex of the human subject [13]. Avian body surfaces also have diverse ecological niches, which likely support distinct microbial communities at different sites. For example, the uropygial gland, located dorsally at the base of the tail, secretes sebum and waxy esters that birds use during preening [14]. Several studies of terrestrial birds indicate that the surrounding nest habitat has a strong influence on the microbiota that are present in the uropygial gland [15, 16]. However, seabirds spend a disproportionately large amount of time preening to make their feathers flexible and waterproof to suit a life at sea [17, 18]. In some bird species, preen oil contains volatile chemical compounds that advertise genetic information, which may be evaluated by conspecifics through odor. For example, in Black-legged kittiwakes (*Rissa tridactyla*), preen oil chemical composition, which may be related to uropygial gland fermentative bacterial communities, correlates with genome-wide heterozygosity [19]. As a result, uropygial gland microbial communities may be individually specific from bird to bird, aiding in recognition and potentially mate selection based on genotypic fitness.

The brood patch is another body site of importance in avian species. This is a highly vascularized ventral body site that enables birds to regulate egg temperature [20] and likely transfers microbial communities to eggs and chicks [21]. This important “first inoculation” of microbes may be comparable to the birth process in mammals, which is known to impact microbial colonization and immune system development later in life [22]. Given these physiological differences, it is likely that the uropygial gland and brood patch sites carry very different microbial communities, but no studies to date have examined the microbiomes of multiple skin locations in any avian species.

While topographical differences in microbiota are likely across bird body sites, between-bird individualized microbiomes may be shaped by several factors. Host genetics is one factor that is known to demonstrably shape microbial communities in other animals [1]. Sex, arguably one of the most important genetic differences, has been shown to impact bacterial communities across a broad range of taxa in humans [23], mammals [7, 9], and birds [24] due to sex-specific behaviors and physiological differences. Relatedness between cellular immune system phenotypes may be another important genetic factor in determining microbiome individuality [25]. Major histocompatibility complexes (MHC), which are suites of highly polymorphic genes associated with immune response in a wide variety of vertebrate taxa [26], are known to influence the composition of gut-associated microbiota in three-spined stickleback fish (*Gasterosteus aculeatus*) [27] and congenic laboratory mice [28] through antigen recognition and activation of humoral and cell-mediated immunity [27, 28]. The frequencies of MHC genotypes in wild populations can be influenced either by disassortative mating or by pathogen pressure [29], but no studies to date have investigated the relationship between MHC and microbiomes in wild birds. This may be particularly important for seabirds, which generally have a lower fecundity, so populations would benefit from a mechanism to detect the underlying genetic makeup of potential mates.

While individual variation plays a role in shaping host microbiota, environmental, and habitat-based factors can also greatly impact bacterial communities. Several studies of genetically similar terrestrial birds indicate that cloacal microbiomes are strongly impacted by the nest in which they were raised [10, 30]. Additionally, microbial communities found on nest material contribute to the composition of the eggshell microbiome in hoopoes (*Upupa epops*) [15] and Reed warblers (*Phragmites australis*) [31], signifying a strong influence of nest environment on bird-associated microbiota. Unlike terrestrial birds, many seabird species spend more time flying over vast ocean water than resting in terrestrial nest environments, and many undertake long annual migrations [32, 33], which may lead to greater exposure to microbial diversity than terrestrial birds. Alternatively, fewer opportunities for seabirds to come in close proximity to the varied microbial habitats in terrestrial environments may limit and homogenize the diversity of microorganisms associated with seabirds.

Finally, factors related to social interactions with conspecifics also help shape individual microbiomes. Within some terrestrial bird species, cloacal bacteria are transferred by allopreening [34] and gut-associated microbiota are more similar between mated pairs [35]. Skin-associated communities can also be influenced by the social interactions. For example, sibling hoopoes reared in the same nest share more uropygial gland bacteria than those reared in separate nests [16]. However, in the off-breeding season, many seabirds lead a more solitary lifestyle than the terrestrial birds on which these studies were conducted, and their microbiomes may be influenced by other environmental determinants.

In this study, we examined the morphological, genetic, environmental, and social factors that contribute to the microbiome of Leach’s storm petrels (*Oceanodroma leucorhoa* or hereafter LESPs). LESPs are an excellent model for microbiome studies. They are categorized within the order *Procellariiformes*, which are highly olfactory bird species. LESPs are known to use odor cues for several important behaviors, including predation, burrow relocation, and identification of conspecifics [36–40]. Because of the importance of olfaction in this species, it is an excellent model for examining microbial differences between individuals, which may relate to volatile organic compound production on the skin [6]. On Bon Portage Island, located off the southern tip of the Canadian province of Nova Scotia in North America, LESPs are abundant, accessible and easily handled, which allows for larger sample sizes than would be possible with other types of olfactory seabirds [41]. These long-lived birds [42] do not participate in extra-pair copulation [43, 44] and tend to return to the same shared burrow each breeding season [45], making it easy to identify a mated pair. They lay a single egg per breeding season that is incubated by one parent while the other forages at sea in shifts that last up to 6 days [42, 46, 47].

Four specific hypotheses were tested with respect to the microbiome of LESPs: (1) Uropygial gland and brood patch sites are colonized by significantly different bacterial communities, (2) genetic diversity, with respect to sex and MHC heterozygosity, influences the composition of bacterial communities at these sites, (3) birds share more bacterial taxa with their own home burrow than with a random non-home burrow, indicating an effect of local habitat on the microbiome, and (4) mated pairs of birds share more bacterial taxa with each other than with non-mates, indicating an effect of social behavior on the microbiome. This is the first study to provide a comprehensive examination of multiple factors that shape the microbiome of a *Procellariiformes* seabird species.

## Methods

### Sample collection

All samples were obtained during the late egg incubation period in summer 2013 (July 18–20) from an established study colony of Leach’s storm petrels located on Bon Portage Island, Nova Scotia, Canada (43° 28′ N, 65° 44′ W) (Additional file 1: Figure S1). Every effort was made to collect samples from as many individual birds as possible in a short time period, so that weather and bird life stage conditions remained consistent. This island is home to an estimated 50,000 breeding pairs of Leach’s storm petrels [48] and over 500 burrows have been mapped across the southern end of the island (G.A. Nevitt unpublished data). Samples for this study were collected from an area of the colony on the landward side of the island where petrel burrows are located among dense balsam fir, red pine, and spruce forest.

Experienced bird handlers removed individuals from their nests and held the birds in place while a research assistant collected swab samples. The skin and feathers near the uropygial glands and brood patches of 22 birds were swabbed for 30 s with sterile cotton swabs (Medline Part#MDS202000) (Additional file 2: Figure S2A). The cotton tip was aseptically broken into a 1.2-mL microfuge tube containing 1 mL of sterile 1× phosphate buffered saline. Swab samples were immediately placed on ice until the end of the field day. Within 8 h of collection, samples were vortexed at high speed for 30 s and centrifuged at 13,000 RPM for 30 min in a microcentrifuge (Eppendorf 5452) and then frozen at − 20 °C. Swab samples were kept frozen during transport to Western Michigan University and were stored at − 80 °C until DNA extraction could be conducted. While the bird was in-hand, a 25 μL blood sample was collected from the isobrachial vein to determine sex and MHC class IIB genotype. The blood was stored in 0.5 mL of Queen’s lysis buffer and shipped to the University of California at Davis for DNA extraction, amplification, and sequencing. Morphometric measurements including bird mass (g), tarsal length (mm), and wing chord length (mm) were recorded.

While the bird was held out of the burrow, soil samples were collected using sterilized 1-cm corers from three locations: deep within the burrow, the entrance of the burrow, and 30 cm away from the burrow entrance (hereafter deep, mid, and surface soils, respectively, Additional file 2: Figure S2B). The time of collection, soil, and air temperature outside the burrow and soil and air temperature inside the burrow were also collected. Soil samples (40 g total) were collected from each of 18 occupied and 7 unoccupied burrows, spread across 43 m in the colony, and subsampled into two Ziploc bags containing 10 g and 30 g of soil each. All soil samples were placed on ice in the field. The 10 g samples were frozen within 8 h of collection, kept frozen during transport to Western Michigan University, and placed in a − 80 °C freezer for storage until DNA extraction could be conducted. The 30 g samples were kept cooled on ice during transport and were used to measure soil abiotic characteristics within 5 days of collection. Once the swab and soil samples were collected, the bird was returned to the burrow (within ~ 5 min) and the sampling team placed a “lattice” of small twigs across the entrance of the burrow. Researchers returned on subsequent days to see if the lattice had been knocked down, indicating that the sampled bird had left the burrow and its mate had returned. In these instances, the mate was also removed from the burrow, and morphological data and blood and swab samples were collected. Samples were obtained from 22 total birds, and it was determined by subsequent genotyping that 14 birds were female and 8 were male. In total, two swabs (uropygial gland and brood patch) were collected from each of the 22 birds; 5 mated pairs were sampled and 25 burrows were sampled (18 occupied and 7 unoccupied controls) at 3 depths per burrow (Additional file 3: Table S1). All samples were stored on wet ice in the field and then frozen on dry ice for transportation prior to analysis. As a control, four additional swab samples were collected and shipped without prior freezing. The communities on these swabs were distinctly different from the others collected in this study, indicating that the frozen storage conditions maintained sample integrity.

### Soil characteristics

For each soil sample, pH, percent soil moisture, nitrate (NO_3_^−^), and ammonium (NH_4_^+^) concentrations were measured on field-cooled soils within 5 days of collection. Large plant roots were removed from all samples prior to taking any measurements. The pH was measured by mixing 5 g of field fresh soil with 10 mL of distilled deionized (DDI) water with a stir bar and recording stable pH using a laboratory meter (Fisher Accumet). Percent soil moisture was determined by placing 10 g of field fresh soil into an aluminum tin and determining the change in mass of soil before and after drying at 65 °C for 1 week. To measure NO_3_^−^ and NH_4_^+^ concentrations, 10 g of fresh soil were shaken at 150 rpm in acid-washed centrifuge tubes with 50 mL of 2 M KCl for 1 h, then centrifuged at 3400 rpm for 5 min and finally filtered through a GF/F filter (Whatman). NO_3_^−^ and NH_4_^+^ concentrations were measured from extracts using 96-well plate protocols [49].

### Bacterial DNA purification

Swab and soil samples were thawed on ice on the day of DNA extraction. DNA was purified using the PureLink Genomic DNA Mini Kit (Life Technologies, Grand Island, New York, K182001) following the manufacturer’s instructions for processing gram-positive bacteria [9, 50]. DNA extracted from swabs was eluted in a final volume of 25 μL of the final elution buffer. DNA from soil microbial communities was purified using the PowerSoil DNA Isolation Kit (Mo Bio Laboratories, Inc., Carlsbad, California, 12888-100) following the manufacturer’s instructions. DNA extracted from soil was eluted in a final volume of 75 μL of elution buffer. Two blank extractions were conducted using each kit to control for contaminant DNA associated with the extractions. DNA was quantified using a Qubit dsDNA HS assay kit (Life Technologies, Q32854) with a Qubit 2.0 quantitation system. DNA concentrations ranged from 10^3^ to 10^4^ ng mL^−1^ for soil and were below detection (< 0.5 ng mL^−1^) for swab extracts. All DNA extracts were stored at − 80 °C prior to library preparation and sequencing.

### Bacterial sequence processing

Amplicon preparation and Mi-Seq (Illumina, San Diego, CA) sequencing was conducted at Michigan State University Genomics Core Facility. Bacterial 16S rRNA genes were PCR amplified using primers specific for the V4 hypervariable region [51]. A subset of PCR products was analyzed on a 1% agarose gel stained with ethidium bromide to ensure that samples contained sufficient DNA for amplification procedures. DNA libraries were normalized using the SequalPrep Normalization Plate Kit, 96-well (Thermo Fisher Scientific, Waltham, MA, A1051001), and samples from each replicate plate were pooled into single wells. Pooled samples were quantified using a Kapa Biosystems qPCR kit (Kapa Biosystems, Inc., Wilmington, MA, KK4824), and samples were normalized to equal concentrations. Each sample pool was spiked with a PhiX control and loaded on an Illumina Mi-Seq flow cell v2 and sequenced using a 500 cycle (PE250) reagent kit in a single sequencing run. Bases were called using Real Time Analysis (RTA) software v1.18.54, and RTA output was de-multiplexed and converted to fastq files using Illumina Bc12Fastq v1.8.4.

Steps for primer sequence removal, quality filtering and merging forward and reverse reads were performed using PANDAseq version 2.8 [52]. Sequences were excluded from analysis if they contained ambiguous base calls, runs of greater than eight identical bases, quality scores of less than 0.9 in a sliding scale of 0 to 1, fewer than 247 bases, more than 275 bases, or sequence overlap of less than 47 bases. After these steps, a total of 8,176,816 high-quality reads remained in the dataset; 518,815 chimeric sequences were identified and filtered with QIIME v.1.9.1 [53] using the USearch 6.1 algorithm [54]. The remaining 7,658,001 sequences were clustered into operational taxonomic units (OTUs) using the pick_open_reference_otus.py script in QIIME, which selected open-reference OTUs via the USearch 6.1 algorithm and removed singleton sequences. Taxonomy was assigned using the Ribosomal Database Project classifier [55] against the Silva version 119 reference database [56]. One thousand seven OTUs were identified in the blank extraction control samples and were removed from the dataset. Three hundred OTUs were identified as associated with Archaea, chloroplasts, and mitochondria, which were also removed from the dataset [51]. After splitting the OTU table by sample type, the resulting swab and soil datasets were rarefied to 9000 and 30,000 sequences per sample, respectively, to equalize sequence reads and reduce bias in community richness and diversity [57, 58]. Following sequence processing, a total of 13 female uropygial gland, 14 female brood patch, 8 male uropygial gland, 7 male brood patch, and 25 burrow samples (deep, mid, and surface soil for each burrow) remained in the dataset (Additional file 3: Table S1). Rarefied datasets were used to conduct downstream comparisons within swab or soil sample types. The entire unrarefied dataset of swab and soil samples combined was used to determine OTUs shared between swab and soil samples.

### MHC genotyping and bird sex determination

MHC genotyping and molecular sexing were conducted at the University of California at Davis. To determine the sex of individual birds, PCR-based protocols (primer pair 2550F and 2718R [59]; primer pair P2 and P8 [60]) were employed to amplify fragments of the chromo-helicase-DNA (CHD) gene in avian sex chromosomes and produce a single fragment in homogametic males (ZZ) or two fragments in heterogametic females (ZW). PCR amplifications were conducted in 26 μL volumes: 90 ng of DNA; 3 pmol of each primer; 13 μL of SYBR Green PCR master mix (Applied Biosystems, 4,309,155) containing 200 μM of each dNTP and 3 mM of MgCl_2_. The thermocycler protocol consisted of an initial denaturation of 95 °C/10 min, 40 cycles of 94 °C/30 s, 48 °C/45 s, and 72 °C/45 s, and a final extension of 72 °C/5 min. When using the P2/P8 primer pair, a restriction digest enzyme (HaeIII) was used to further cleave the amplified W fragment, allowing for an easier discrimination between fragment sizes. All PCR products were checked using 2% agarose gels (TAE: 400 mM Tris, .01 M EDTA, pH 8.3) and stained with ethidium bromide or SYBR-Safe (Invitrogen, S33102) DNA gel stain.

To identify the MHC genotypes of individual birds, locus-specific primers were developed from a previous characterization of one MHC class IIB genomic fragment (OcleDAB2Fw or DAB2; primer sequences and PCR protocol described in [61]). Concurrent research suggests that this locus is strongly under selection [43]. Gene fragments 300 bp in size were amplified at this locus and fragments were sequenced using BigDye 3.1 technology and ABI3130xl/ABI3730 automated sequencers (University of California Davis Gene Sequencing Center). Sequence chromatograms were aligned using BioEdit sequence alignment editor [62].

#### Statistical analyses

A summary of statistical analyses used in this study can be found in Additional file 4: Table S2. Observed OTUs were used as a measure of community richness to calculate within-sample alpha diversity based on the Shannon-Weaver index [63] using R version 3.3.0 [64], implemented through R Studio version 0.99.902 [65], and vegan version 2.3-5 [66]. Differences in Shannon diversity between groups was determined using Wilcoxon rank-sum tests. Between-sample beta diversity was calculated using the weighted UniFrac distance matrix, which generates pairwise distances based on species abundance and phylogenetic branch length [67]. Multivariate data were visualized using the two most explanatory axes of a principal coordinates analysis (PCoA) using PhyloSeq version 1.16.2 [68]. Non-parametric permutational analysis of variance (PERMANOVA) tests for group significance within multivariate community data and was determined using the adonis function in vegan. OTUs responsible for between-group differences were determined by simper (similarity percentage) analysis [69], which included OTUs that contributed to at least 70% of the differences between groups of interest. The envfit function in vegan was used to overlay morphological and genetic vectors on appropriate PCoA plots, and differences in percent shared OTUs between birds and burrow soil sites were determined by one-way ANOVA. Samples for which complete morphological data was not available were removed from envfit analyses. Differences between groups were also compared based on bacterial OTU relative abundances. Between-sex comparisons of bacterial community relative abundances at the phylum and family level were performed on datasets transformed using the powerTransform function in the library car [70] by one-way ANOVA [71], and orthogonal contrasts were performed on groups of interest using the package Phia [72]. Abiotic soil properties were compared among deep, mid, and surface soil from occupied and unoccupied burrows using two-way ANOVA, and orthogonal contrasts were performed on groups of interest. One-way comparisons of non-normal datasets were analyzed using Kruskal-Wallis tests [73]. Distance matrices based on burrow coordinates were generated using the spDists command in the sp v1.2-3 package [74], and mantel tests were used to determine correlations between UniFrac and burrow distance matrices [75]. For each female to male comparison, females were compared to the mated male and a randomly assigned non-mated male. Welch’s two-sample *t* tests [76] were used to compare percent shared OTUs between birds in mated pairs and between females and randomly assigned males.

## Results

### Individual effects on petrel-associated microbiota: influence of sex, morphology and genetics

**Fig. 1:**
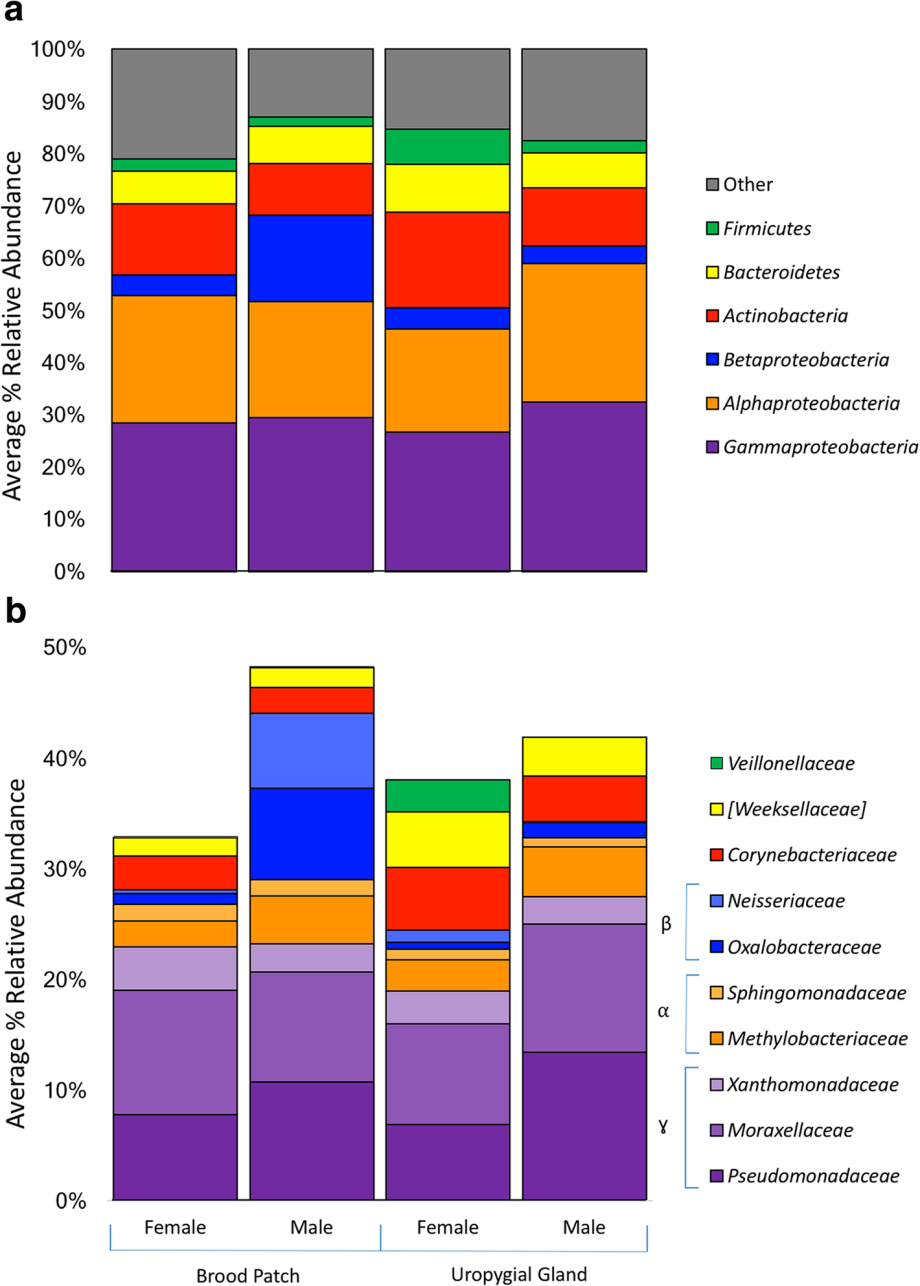
Relative abundance of bird-associated bacterial communities by phylum (**a**) and most abundant families (**b**). Both body sites were characterized by highly abundant *Proteobacteria*, *Bacteroidetes*, *Firmicutes*, and *Actinobacteria*. Families represent the top 20 most abundant OTUs. Colors of the families in **b** correspond to the phyla represented in **a**. *Proteobacteria* are marked as (β) *Betaproteobacteria*, (α) *Alphaproteobacteria*, and (γ) *Gammaproteobacteria*. Remaining families not represented in this figure are listed in Additional file 9: Table S4

Bacterial communities varied by both swab location and sex of the bird, so all analyses were conducted categorically to avoid confounding results (Additional file 5: Figure S3). Categories are female brood patch swabs, female uropygial gland swabs, male brood patch swabs, and male uropygial gland swabs, as described in Fig. 1. Within each sex, observed OTU richness was similar between the uropygial gland and brood patch body locations. The same six phyla or sub-phyla represented the greatest relative abundance in all swab communities (*Gammaproteobacteria*, *Alphaproteobacteria*, *Betaproteobacteria*, *Actinobacteria*, *Bacteroidetes*, and *Firmicute*s, Fig. 1a). Other phyla represented < 20% of the total relative abundance for each swab site category. At a finer scale of resolution, the main families of bacteria represented at each body site and on both sexes were also highly similar (Fig. 1b), but with key differences. *Pseudomonadaceae*, *Moraxellaceae*, *Xanthomonadaceae*, *Methylobacteriaceae*, *Sphingomonadaceae*, *Oxalobacteraceae*, *Neisseriaceae*, *Corynebacteriaceae*, *Veillonellaceae*, and candidate family *Weeksellaceae* were all represented in 0.01–13.4% relative abundances in each sample category. However, total within-sample alpha diversity, including both swab locations, was higher in female birds (Shannon index, 4.66 ± 0.181) than male birds (Shannon index 4.17 ± 0.356). This difference was driven by significantly higher diversity at female brood patches (Wilcoxon rank-sum test, Shannon index, *W* = 79, *p* = 0.025) than the male brood patches, but uropygial sites had similar diversity in both sexes (*W* = 72, *p* = 0.162). Between-sex bacterial communities were structurally different at the brood patch sites (pseudo-F = 1.5884, *p* = 0.03, *n* = 21) and uropygial glands (pseudo-F = 2.3323, *p* = 0.005, *n* = 21). Simper analysis, which identifies OTUs that differ most in relative abundance between samples, indicated that OTUs within the families *Pseudomonadaceae*, *Moraxellaceae*, *Corynebacteriaceae*, *Methylobacteriaceae*, and *Sphingomonadaceae* were most responsible for the structural differences observed in uropygial gland communities between males and females. In contrast, bacteria within the families *Neisseriaceae*, *Pseudomonadaceae*, *Methylobacteriaceae*, *Oxalobacteraceae*, and *Moraxellaceae* drove differences between male and female brood patch communities (Table 1).

**Table 1:** OTUs identified using SIMPER analyses most responsible for bacterial community differences between males and females at the uropygial gland and brood patch

Comparison	Five most influential OTUs	Represented Family	% contribution to difference	% average abundance (female)	% average abundance (male)
Female vs. male, Uropygial gland	KC358339.1.1270	*Pseudomonadaceae*	3.1	4.82 ± 0.011	8.37 ± 0.015
	FJ612285.1.1489	*Moraxellaceae*	2.4	1.99 ± 0.018	5.20 ± 0.025
	CP001809.1856259.1857766	*Corynebacteriaceae*	2.1	5.04 ± 0.008	4.04 ± 0.011
	JF222412.1.1310	*Methylobacteriaceae*	1.7	2.48 ± 0.009	4.06 ± 0.013
	FJ891018.1.1343	*Sphingomonadaceae*	1.7	2.14 ± 0.005	4.69 ± 0.007
Female vs. male, Brood patch	JQ191134.1.1362	*Neisseriaceae*	3.2	0.16 ± 0.039	6.23 ± 0.056
	KC358339.1.1270	*Pseudomonadaceae*	2.7	4.79 ± 0.011	4.94 ± 0.016
	JF222412.1.1310	*Methylobacteriaceae*	2.0	1.97 ± 0.014	3.97 ± 0.020
	JQ316675.1.1495	*Oxalobacteraceae*	1.9	0.06 ± 0.020	3.85 ± 0.028
	FJ612285.1.1489	*Moraxellaceae*	1.8	2.43 ± 0.010	3.34 ± 0.013

**Fig. 2:**
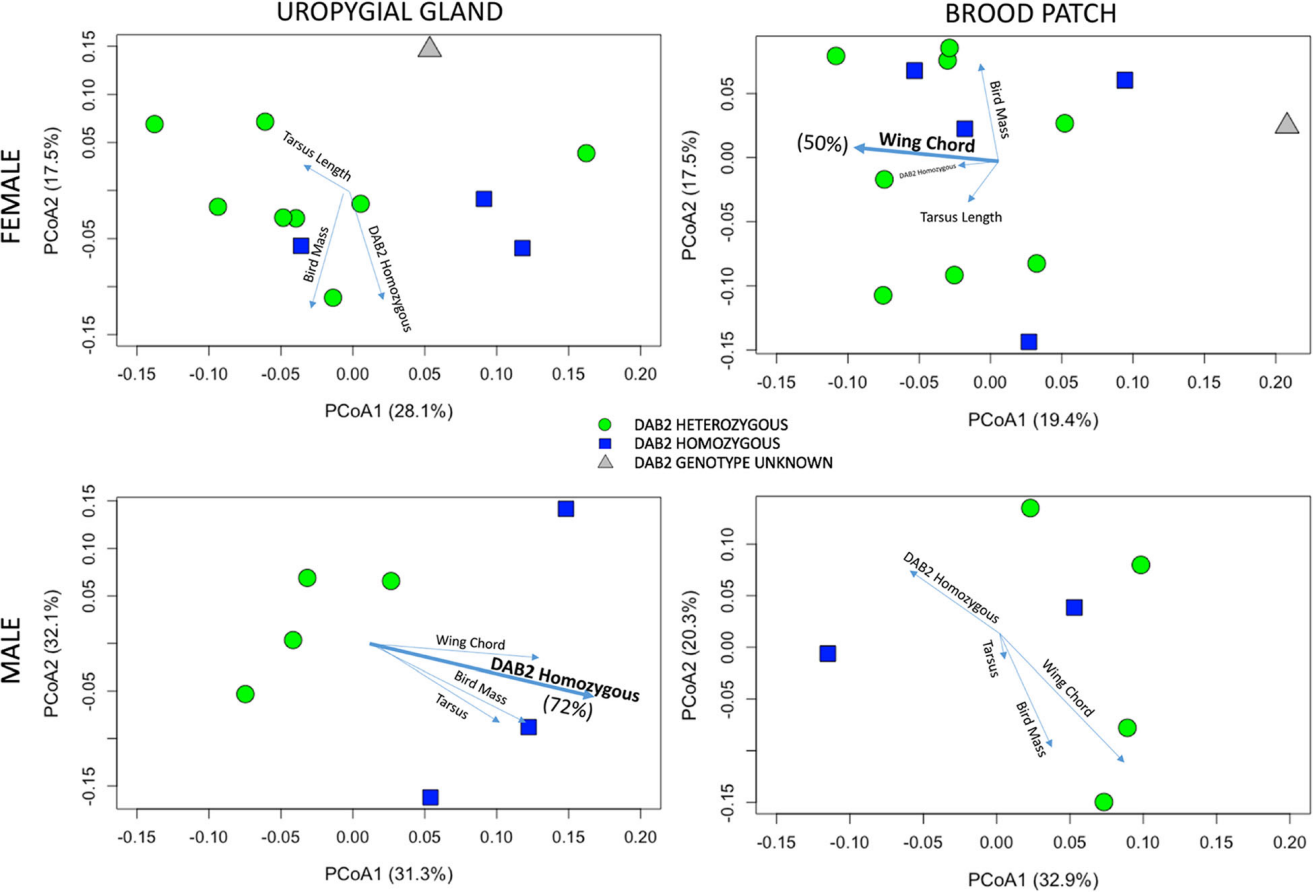
PCoA of female uropygial gland, female brood patch, male uropygial gland, and male brood patch bacterial communities based on weighted UniFrac dissimilarity. Green circles represent DAB2 heterozygous individuals, blue squares represent DAB2 homozygous individuals, and gray triangles represent individuals lacking genotype data. Morphological and genetic factors are represented by arrows, and the length of each arrow is proportional to the explanatory power of each variable. Female wing chord length explained 50% of variation in brood patch bacterial community structure (*R* ^2^ *=* 0.500, *p* = 0.024, *n* = 14). DAB2 homozygosity explained 72% of variation in male uropygial gland community structure (weighted UniFrac pseudo-F = 1.859, *p* = 0.015, *n* = 8), although sample size was small for this analysis. Wing chord was too small to represent for female uropygial gland analysis

In this population of LESPs, only one morphological characteristic (wing chord length) varied by sex. Female birds had longer wing chords than males (females, 162.72 ± 1.56 mm; males, 159.59 ± 2.10 mm, *t* = −2.657, *p* = 0.02), but did not differ by mass (females, 48.51 ± 2.42 g; males, 50.47 ± 2.61 g, *t* = −1.138, *p* = 0.268) or tarsus length (females, 24.42 ± 0.41 mm; males, 24.58 ± 0.49 mm, *p* = 0.560). Wing chord length explained a significant amount of variation in female brood patch bacterial community structure (*R* ^2^ *=* 0.53, *p* = 0.024, *n* = 13; Fig. 2), indicating that birds with longer wing chords carried more similar microbiota. No other morphological parameters correlated with brood patch community structure in either males or females.

While wing chord length explained some variation in female microbiota, MHC genetics explained significant amounts of variation in bacterial community structure at the male uropygial site (Fig. 2). In LESPs, the DAB2 gene expresses an MHC class II antigen which aids in immune system function. Bacterial community structure at the uropygial gland differed between males that were homozygous and heterozygous at this gene locus (weighted UniFrac, pseudo-F = 1.859, *p* = 0.015, *n* = 8), and DAB2 homozygosity significantly correlated with bacterial community structure (*R* ^2^ = 0.718, *p* = 0.048, *n* = 8; Fig. 2). However, in females, allele identity at DAB2 did not influence bacterial community structure at the uropygial gland (weighted UniFrac, pseudo-F = 1.100, *p* = 0.339). Additionally, DAB2 heterozygosity did not explain any variation in bacterial community structure at the brood patch location in either sex (*p* > 0.319).

### Environmental effects on petrel-associated microbiota: influence of burrow soil and oceanic bacteria

Each mated pair builds and inhabits an underground burrow and may acquire microbiota from the environment. Soil abiotic factors and bacterial communities were analyzed from three depths of each petrel burrow (deep, mid, and surface)**.** Bacterial communities collected from all depths did not differ between burrows that were occupied by a bird and those that were unoccupied during the sampling season (*p* = 0.262, *n* = 18 occupied/7 unoccupied, Additional file 6: Figure S4). Additionally, soil pH, NH_4_^+^ concentrations, and moisture contents were similar between occupied and unoccupied burrows at each of the three depths (Additional file 7: Figures S5A–S5C). A distinguishing microbiome of a specific home burrow was not reflected in the microbiome of its occupant(s). Birds shared the same amount of OTUs with their home burrows as with a randomly selected non-home burrow, regardless of sex or body site location (*p* > 0.05) and despite a significant geographic variation in burrow soil communities across the burrows sampled (Additional file 8: Table S3). Burrow soil bacterial communities differed by depth, and by averaging all values at all body sites, birds shared the greatest amount of OTUs (4.6 ± 0.98%) with the microbial community in the deep burrow soil samples, where the nest is located (Table 2, *p* = 0.02, *n* = 22). Male and female birds shared a similar percentage of OTUs with deep burrow soil regardless of body site (*p* > 0.5, *n* = 22).

**Table 2:** OTUs shared between birds and their burrow environments. Significance was determined by using Welch’s two-sample *t* test. Birds shared on average 4.6% (± 0.98) OTUs with deep burrow soil and did not share more with their own burrow environment than with a randomly chosen “away” burrow (*p* > 0.05)

Comparison	Burrow soil site	Test statistic *t*	*P* value	Burrow	Mean % shared OTUs	95% CI	*n*
Female uropygial gland	Deep	0.135	0.894	Home	4.53	2.54	13
				Away	4.59	2.24	13
	Mid	0.667	0.513	Home	3.32	1.39	13
				Away	3.33	1.43	13
	Surface	0.008	0.994	Home	3.08	1.37	13
				Away	3.15	1.58	13
Female brood patch	Deep	− 0.470	0.644	Home	5.25	1.94	14
				Away	5.62	2.73	14
	Mid	− 0.404	0.691	Home	4.36	1.63	14
				Away	4.07	1.43	14
	Surface	− 0.177	0.861	Home	3.81	1.26	14
				Away	3.86	1.62	14
Male uropygial gland	Deep	0.455	0.673	Home	3.79	1.63	8
				Away	3.60	2.40	8
	Mid	− 0.748	0.489	Home	2.99	2.22	8
				Away	3.05	2.22	8
	Surface	− 0.482	0.653	Home	2.53	1.57	8
				Away	2.49	1.71	8
Male brood patch	Deep	− 0.238	0.820	Home	4.22	1.64	7
				Away	3.63	1.57	7
	Mid	0.342	0.748	Home	3.00	1.61	7
				Away	3.07	1.63	7
	Surface	0.457	0.667	Home	2.48	1.03	7
				Away	2.61	1.22	7

Since LESPs spend most of their time in flight over the ocean or interacting with marine water to forage, oceanic microorganisms may also be an environmental source of diversity to their microbiome. During incubation, LESPs travel up to 1000 km and can be gone at sea for over 6 days before returning to the nest [47]. It was hypothesized that LESPs would carry a high percentage of distinctive ocean bacteria due to these long foraging trips. Taxa that have been identified as ocean-associated, including members of *Rhodospirillaceae*, *Burkholderia*, and *Microbacteriaceae* [77] were detected on the birds sampled in this study (Table 3). Ocean-associated taxa constituted an average of 6.53 ± 0.11% of the total bacteria detected on LESPs, indicating that this source of bacterial diversity is possibly a more important determinant of the petrel microbiome than the terrestrial burrow. Also, methodological limitations prevent the identification of bacteria at the species level, so this percentage is likely higher. There were no significant between-sex or within-sex abundance differences at either skin site for any of the ocean taxa analyzed (*p* > 0.05).

**Table 3:** Bacterial genera detected on female and male LESPs. Ocean-associated bacteria constituted only 6.53% ± 0.11 of the total bacteria detected on LESPs. There were no significant between-sex or within-sex relative abundance differences at either skin site for any of the ocean-associated taxa analyzed (*p* > 0.05)

	Average % relative abundance			
Classification	Female brood patch (*n* = 14)	Male brood patch (*n* = 7)	Female uropygial gland (*n* = 13)	Male uropygial gland (*n* = 8)
*Chlorobi*	0.042 ± 0.067	0.000	0.000	0.000
*Cytophagia*	0.006 ± 0.004	0.062 ± 0.059	0.007 ± 0.006	0.001 ± 0.002
*Bdellovibrionales*	0.011 ± 0.013	0.001 ± 0.001	0.003 ± 0.003	0.002 ± 0.003
*Burkholderiales*	0.008 ± 0.002	0.022 ± 0.018	0.006 ± 0.003	0.007 ± 0.003
*Rhodospirillaceae*	0.006 ± 0.003	0.001 ± 0.001	0.006 ± 0.003	0.007 ± 0.004
*Cellulomonadaceae*	0.006 ± 0.012	0.000	0.006 ± 0.008	0.000 ± 0.001
*Microbacteriaceae*	0.014 ± 0.013	0.005 ± 0.004	0.041 ± 0.015	0.016 ± 0.010
*Synechococcus*	0.000	0.062 ± 0.123	0.000 ± 0.001	0.000

### Social effects on petrel-associated microbiota: influence of mate microbiota

It was hypothesized that social interactions between the two individuals in a mated pair, as well as their co-habitation of the same burrow, would also play a role in structuring the microbiome. However, when microbiota of the two individuals in mated pairs were compared to each other at both body sites, there was no significant difference in the amount of shared OTUs between a female and its male burrow mate and non-mates (*t* = − 1.767, *p =* 0.100, *n* = 9, Table 4). Additionally, there was no significant difference in the amount of shared OTUs between a male and its burrow mate and non-mates (*t* = 0.323, *p* = 0.752, *n* = 8). A lack of significance was observed while comparing body sites together or separately (Table 4).

**Table 4:** OTUs shared between birds and their burrow mates. Significance was determined using Welch’s two sample *t* test. Birds did not share more OTUs with their burrow mates than with a randomly selected non-mate (*p* > 0.05)

Swab type	Test statistic *t*	*P* value	Bird	Mean % shared OTUs	95% CI	*n*
Female all samples	− 1.7666	0.1001	Burrow mate	10.57	1.03	9
			Random	12.06	1.65	9
Male all samples	0.32337	0.7518	Burrow mate	10.56	1.19	8
			Random	10.23	1.72	8
Female uropygial gland	− 0.91587	0.4025	Burrow mate	11.13	2.12	4
			Random	12.32	3.54	4
Female brood patch	− 1.4571	0.1935	Burrow mate	10.11	1.60	5
			Random	11.85	2.88	5
Male uropygial gland	− 0.53481	0.6335	Burrow mate	11.29	3.94	3
			Random	11.83	1.69	3
Male brood patch	0.71256	0.5002	Burrow mate	10.11	1.60	5
			Random	9.34	2.58	5

## Discussion

In general, the skin microbiome of birds has been under-evaluated and is supported by only a handful of studies that examined differences in microbial communities across body sites [10, 78, 79]. In this study, 16S rRNA amplicon sequences were used to demonstrate that bacterial communities in LESPs were body site and sex specific. Male and female brood patches and uropygial glands harbored significantly different bacterial communities. While several terrestrial bird microbiome studies have been conducted, this is the first examination of the external microbiome of a seabird and the first study to include the avian brood patch as a site of investigation.

Feathers and skin are particularly of interest because they are the first barrier between a bird’s body and the environment. As such, interactions between birds, their conspecifics and their nest environments are likely to influence the composition of the skin microbiome [10, 79]. A thorough understanding of this phenomenon may play a crucial role in the next generation of wildlife disease protection. For example, in protected wild bird populations, microbiome monitoring could be used as a warning sign for increases in bird-specific pathogen outbreaks, such as feather-destroying *Bacillis licheniformis*, and its potential transfer from brood patch to egg shell during incubation. By understanding more about bird microbiota, predictive tipping points in microbial communities of at-risk bird species or economically important recreational bird species could aid in proactive prevention of damaging pathogenic disease.

### Individual effects on petrel-associated microbiota: petrel microbiota differ by body site and sex

Core taxa colonizing the uropygial gland and brood patch sites of LESPs belonged to the phyla *Proteobacteria*, *Bacteroidetes*, *Firmicutes*, and *Actinobacteria*. At this taxonomic level, these results were similar to previous studies investigating the microbiomes of other bird species at multiple body sites [10, 80]. However, when examined at a higher level of resolution, uropygial gland and brood patch bacterial communities were very different, especially across individual females and between sexes. Bacteria within *Alphaproteobacteria* and *Gammaproteobacteria*, including ecologically important *Pseudomonadaceae* and *Methylobacteriaceae*, were predominant community members at the uropygial glands of both sexes, though males carried more. *Pseudomonas* are known odor producers, capable of using oils as substrates to produce volatile organic compounds, or VOCs [81], and members of *Methylobacteriaceae*, while common in the environment, are associated with human foot odor [82]. The presence of these two known odor-producing bacterial families at the uropygial gland site suggests that sex-specific bacterial production of VOCs may play an important role in olfactory communication in LESPs. Bacteria belonging to another odor-producing family, *Corynebacteriaceae*, were abundant in the uropygial glands of both sexes, but especially in females. This family of bacteria is known to metabolize apocrine sweat to produce VOCs on human bodies to produce odor [13, 83]. This difference in known odor-producing bacterial taxa between males and females may be related to odor-based cues in olfactory-mediated behavior between and among the two sexes.

The waxy, sebaceous microenvironment of the uropygial gland and the seasonally bare and warm environment of the brood patch [84] provide fundamentally different ecological niches for microbiota. The uropygial gland secretes lipids and sebum that birds spread over their plumage during preening [14, 18]. The distinctive uropygial gland microenvironment likely selects for subsets of microbiota, a phenomenon seen in other animal species. For example, sebum production, pH, and moisture differ among human skin sites and lead to fundamentally different bacterial communities at different locations [13]. Similarly, in dogs, haired skin sites support the growth of different bacterial communities than mucosal skin sites [85].

Unlike the uropygial gland sites, brood patch sites were significantly more diverse in female LESPs than males. The brood patch is a small, hypervascularized portion of skin that comes into direct contact with the egg to regulate appropriate incubating temperatures [20, 86], and sex-specific differences at this site occur in many avian species. There are several physiological differences between male and female brood patches, even in bird species where both sexes, including LESPs, contribute to incubation activities [20]. For example, in Zebra finches (*Taeniopygia guttata*), the female brood patch transfers more heat to the egg than the male brood patch [87]. Male Reed warblers (*Acrocephalus scirpaceus)* increase egg temperature during incubation at a faster rate than females [88] and male Yellow-eyed penguins (*Megadyptes antipodes*) have higher brood patch temperatures than females [89]. While unstudied in LESPs, variation in temperature ranges between male and female brood patches is a possible contributor to microbial variation at that location. In this study, males and females carried the same core taxa at brood patch sites, but those taxa varied in relative abundance. Male brood patches harbored more *Pseudomonadaceae* and *Methylobacteriaceae*, while females carried more *Moraxellaceae*. All of these families contain species of bacteria that are implicated in odor production [81, 82, 90]. However, further study is required to definitively connect variation in brood patch temperatures, sex, and odor production in any bird species.

Many sex-specific physiological differences can interact to impact bacterial communities. One example is that fluctuations in reproductive hormone levels occur over time with respect to reproductive state, which can have drastic effects on bacterial community structure in vertebrates [7, 9]. This phenomenon also applies to birds. In the Wandering Albatross (*Diomedea exulans*), a close relative of the LESP, estradiol and testosterone levels increase prior to egg laying and then drop off sharply during early incubation [91]. This also occurs in Black kites (*Milvus migrans*) and Canvasback ducks (*Aythya valisineria*) [92, 93]. While it was beyond the scope of the current study to measure hormone levels, samples were collected at a time immediately following when female petrels had produced eggs and most of the nests contained eggs at the time of sampling. If hormonal fluctuations for LESPs are similar to those studied in other bird species, then estradiol levels in female LESPs at the time of sampling may have been reduced compared to pre-laying levels. This reduction may have been a factor contributing to fewer detected *Pseudomonas* spp., which are known to be sensitive to estradiol [94, 95]. Additionally, males are more susceptible to some *Pseudomonas* spp. due to a suppressive effect of testosterone on the immune system [96]. Correlations between hormone fluctuations and microbiome composition have been reported in non-avian species, including meerkats [9], hyenas [7], and humans [97]. However, sex-specific differences are not always observed. Contrary to consensus findings, a recent study demonstrated that female and male Dark-eyed juncos shared similar cloacal and uropygial microbial communities [10]. However, those birds were sampled close to the time nestlings fledged, likely allowing hormone levels adequate time to return to post-reproductive levels, which may have altered bacterial community structure. Thus, in LESPs, it is possible that hormonal status in females could have been one contributor to differences between male and female microbiota.

Additionally, sex-specific behaviors, which occur in many bird species, could contribute to structural differences between female and male bacterial communities. The closely related European storm petrel exhibits sex-specific migratory patterns [98] which may have implications for host exposure to the environment, food intake, and subsequent effects on microbial community structure. Female Wilson’s storm petrels take longer trips and provide heavier meals to chicks during times of food scarcity, which can increase environmental exposure to more diverse microorganisms in females [99]. In this study, LESP males carried more OTUs associated with *Oxalobacteraceae* and *Methylobacteriaceae* than females. Members of both taxonomic groups are commonly found in the environment, often associated with the plant phyllosphere, rhizosphere, and soil [100, 101]. The differential abundance of these bacterial families on male and female birds suggests that differing interactions of LESPs with their burrows may also be a contributor to overall sex-specific differences in microbiota.

### Individual effects on petrel-associated microbiota: MHC genotype may influence the microbiome in a site- and sex-specific manner

While sex-specific differences impacted bacterial community structure at multiple body sites, genetic factors associated with MHC genotype also explained some variation in bacterial communities. The MHC is a class of highly polymorphic immunogenetic markers, whose variability in natural populations is maintained by pathogen-mediated selection, disassortative mate choice, and maternal-fetal effects [25]. In some organisms, MHC appears to influence individual body odor, possibly due to interactions with microbial communities [6]. For example, in black-legged kittawakes, chemicals found in preen secretions correlate positively with MHC relatedness [102]. Additionally, microbiomes have been shown to differ by MHC genotypes in non-avian species, including the three-spine stickleback fish (*Gasterosteus aculeatus*) and inbred laboratory mice [27, 28]. In this study, the results show that male LESPs carrying homozygous DAB2 genotypes had significantly different uropygial gland bacterial communities than heterozygous males. Mate choice in this species may be in part mediated by olfaction [103] and assessment of individual odors, which may be influenced by odor-producing microbiota. However, it is important to note the small sample size associated with this result (*n* = 8), particularly with respect to the population-level diversity of MHC genotypes in LESPs (i.e., there are 16 identified alleles for DAB2 alone). A much larger assessment of this population is necessary to tease apart the complexities of all allelic combinations and microbiome diversity and to verify this result with a larger sample size.

### Environmental effects on petrel-associated microbiota: petrel and burrow microbiomes share little OTU overlap

Although individual-specific variation impacted bacterial community structure, LESPs spend considerable time in the burrow during egg incubation, which might lead to specific environmental effects of the burrow on bird-associated microbiota. Although burrow-associated microbial communities co-varied with geographical burrow distance, bird-associated communities did not. The results from this study demonstrated that only 4.6% burrow-associated OTUs overlapped with the LESP microbiome. This result is contrary to findings in terrestrial birds, where nest environment significantly correlated with bacterial community composition [30]. Many of the OTUs shared between birds belonged to families that were abundant in the soil environment (Additional file 6: Figure S4B). For example, OTUs in the families *Acidobacteriaceae* and *Koribacteraceae* are ubiquitously found in soil [104], and members of *Hyphomicrobiaceae*, belonging to the highly diverse *Alphaproteobacteria*, were found in both soil and bird samples [105, 106]. *Xanthomonadaceae* species, particularly denitrifying *Rhodantobacter* spp., are common environmental bacteria [107, 108] that were also in high abundances in the microbiome. Despite these overlaps, the burrow bacteria contributed relatively little to LESP microbiomes.

In contrast, 6.53% of bacteria found on LESPs were identified as ocean-associated taxa. While still a low percentage, this indicates that the marine environment is likely an equal or greater contributor to the microbiome of LESPs than burrow soil and does not account for marine-associated bacterial species that have yet to be discovered [109]. LESPs often make long foraging trips to sea that can last up to 6 days and acquire food at the ocean surface, where they are exposed to ocean water and marine bacteria. However, both environmental factors contributed relatively little to the total diversity of the LESP microbiome, while individualistic and sex-specific factors play a strong role in describing the variation in microbiomes across individuals.

### Social effects on petrel-associated microbiota: petrel microbiomes are not influenced by social interactions between mated pairs

Socially monogamous pairs of LESPs share a common burrow environment, lending support to the idea that birds in a mated pair would be expected to share bacterial communities. However, in this study, LESPs shared the same amount of OTUs with their burrow mates as with randomly chosen non-mates (10.56 ± 1.03), which is more than double the amount of OTUs shared between individual birds and their burrows. While the sample size for this observation is low (*n* = 5 mated pairs), this result is contrary to several studies that show much stronger effects of social interactions in terrestrial bird species. For example, heterospecific Great tits (*Parus major*) raised in the same nest had more similar cloacal microbiomes than biological siblings reared in separate nests [30], and Dark-eyed junco nestlings had more cloacal microbial taxa in common with their mothers than with their fathers due to frequency of physical contact [10]. There are several possible reasons for these disparate observations. First, in contrast to many other bird species, LESP mates rarely occupy the burrow at the same time after egg laying. While one bird remains in the burrow with the egg, its mate spends several days away foraging for food at sea [42]. As a result of this behavior, few bacteria would be shared between the two birds if they have limited physical contact. Second, female petrels had more OTUs in common with other females, and male petrels had more in common with other males, than either sex had with its mate. Here, sex-specific factors were more important in describing the LESP microbiome than interactions with the mate, and further studies with a larger sample size are required to determine whether any meaningful exchange of microorganisms occurs between mated pairs or between parents and offspring.

## Conclusions

This investigation is the first study to provide information about the factors that influence the external microbiome of a unique migratory seabird that spends most of its life at sea, lives in nests underground, and relies heavily on olfaction for critical life activities. The results demonstrate that sex and body site play the most important roles in defining the LESP microbiome. MHC genotype also explained variation in male uropygial gland communities. In contrast to terrestrial bird species, very little influence of the environment or social interactions was observed with respect to LESP microbiomes. This lack of environmental and social influence is likely indicative of the LESP lifestyle. LESP-mated pairs spend little time together in a burrow and travel over 1000 km per trip to forage for food in the ocean [47]. As a result, LESP microbiota are much more driven by individualistic determinants than other bird species that have been studied. The novel results demonstrated here show that body site location and sex are more influential than the environment on the microbiome of these seabirds. Unlike laboratory studies of animal microbiomes, the sample number in this study depended upon timing and the ability to collect samples from animals in the wild. As a result, the sample size for certain tests, such as comparison of MHC alleles and comparisons between mated pairs, is less than ideal and possibly subject accepting false negatives (type II error). Further targeted studies that use an experimental approach are necessary to determine whether any of the 16 MHC allele combinations in LESPs result in different microbiome compositions, but this type of study would require an enormous sampling effort to ensure that birds carrying all allele combinations were represented and replicated. Similarly, further studies are also required to determine whether these results are exemplified by all migratory seabird species. In general, assessments of wild bird-associated microbiota are important for understanding health, preservation, and behavior, to inform management and pathogen protection activities. Additional connections between the microbiome and olfactory communication within *Procellariiformes* species will have an enormous impact on further understanding the link between the microbiome, its influence on chemical sensing, and mate selection. The results of this study add a unique perspective to this knowledge base, demonstrating that LESP microbiomes are more strongly shaped by intrinsic genetic factors and less impacted by environmental interactions.

## Additional files

Additional file 1: Figure S1. Map of sampling locations on Bon Portage Island, Nova Scotia, Canada. Study samples were collected from an area of the colony (approximately 4 m^2^) where petrel burrows are located among dense balsam fir, red pine, and spruce forest. (DOCX 217 kb)

Additional file 2: Figure S2. Diagram of bird body sampling locations and burrow soil sampling depths. (DOCX 136 kb)

Additional file 3: Table S1. Sampling summary. Two swab samples (uropygial gland and brood patch) were collected from each of 22 birds. Genotyping determined that 14 birds were female and 8 were male, and 5 male/female dyads were mated pairs. Burrow soil was sampled at 3 depths per burrow. (DOCX 20 kb)

Additional file 4: Table S2. Summary of statistical analyses. Statistical analyses were performed using R, and each command and R package used is specified for each analysis. (DOCX 106 kb)

Additional file 5: Figure S3. Principal coordinates of analysis of bird-associated bacterial community structure. Bacterial communities varied by both body site and sex of the bird. The sex of the bird had a strong influence on bacterial community structure at the uropygial gland and brood patch. Female birds carried different microbial communities at each of the two body sites examined, but body sites in male birds did not have different bacterial communities. Based on these results, all analyses were conducted categorically to avoid confounding results. (DOCX 320 kb)

Additional file 6: Figure S4. Relative abundance of top 25 bacterial species ranked by phylum and family among occupied and unoccupied deep, mid, and surface burrow soil categories. Burrow occupancy had no effect on bacterial community composition or structure, but burrow communities were significantly different based on depth. (DOCX 293 kb)

Additional file 7: Figure S5. Soil abiotic properties between 18 occupied and 7 unoccupied burrows at deep, mid, and surface burrow soil. Soil pH was significantly lower in deep burrow soil, and NH_4_^+^ concentration was significantly higher in deep burrow soil compared to surface burrow soil. Soil moisture was similar between occupied and unoccupied burrows and was similar at all soil depths, while burrow occupancy had no effect on soil pH or soil moisture. (DOCX 153 kb)

Additional file 8: Table S3. Comparison of burrow soil distance matrices with geographical distance. Weighted and unweighted UniFrac discance matrices were compared to geographical burrow distance. At all burrow soil depths, community presence/absence significantly correlated with geographical burrow distance and bacterial community structure from mid and surface burrow soil significantly correlated with geographical burrow distance. (DOCX 17 kb)

Additional file 9: Table S4. Families represented within top phyla. In Fig. 1b, families representing the top 20 most abundant species are shown. The remaining families within each phylum are shown in Fig. 1 are listed in this table. (DOCX 121 kb)

## Abbreviations

LESP: Leach’s Storm petrel
