# Supplementary material for: Morphological and genetic factors shape the microbiome of a seabird species (Oceanodroma leucorhoa) more than environmental and social factors
Source: Microbiome. 2017 Oct 30;5:146. doi: 10.1186/s40168-017-0365-4 (PMC5663041; doi:10.1186/s40168-017-0365-4)
Supplement: Supplementary file 1 — Map of sampling locations on Bon Portage Island, Nova Scotia, Canada. Study samples were collected from an area of the colony (approximately 4 m2) where petrel burrows are located among dense balsam fir, red pine, and spruce forest. (DOCX 217 kb) [file 40168_2017_365_MOESM1_ESM.docx]

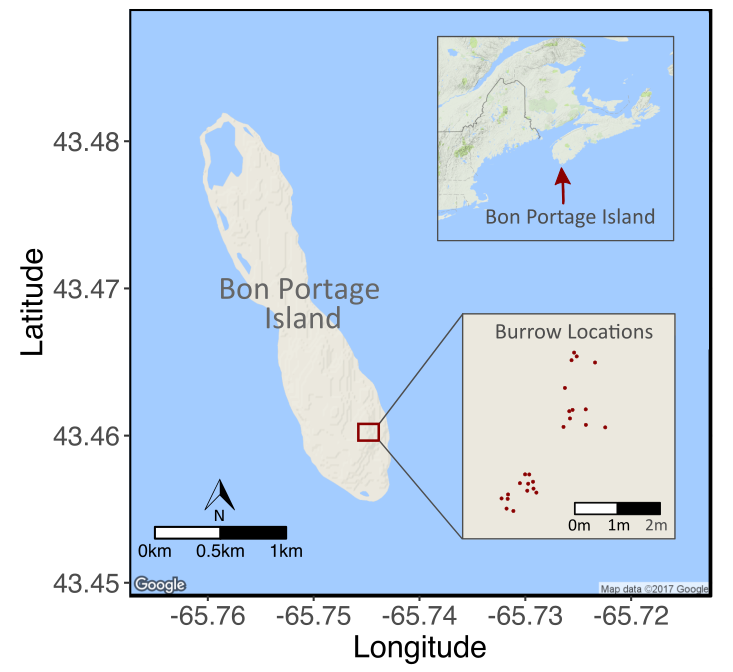


**Figure S1**- Map of sampling locations on Bon Portage Island, located off the southern tip of Nova Scotia, Canada. Distance between burrows is estimated with inset scale. This island is home to an estimated 50,000 breeding pairs of Leach’s storm-petrels and over 500 burrows have been mapped across the southern end of the island. Samples for this study were collected from an area of the colony (approximately 4 m^2^) where petrel burrows are located among dense balsam fir, red pine and spruce forest.
