# Supplementary material for: Morphological and genetic factors shape the microbiome of a seabird species (Oceanodroma leucorhoa) more than environmental and social factors
Source: Microbiome. 2017 Oct 30;5:146. doi: 10.1186/s40168-017-0365-4 (PMC5663041; doi:10.1186/s40168-017-0365-4)
Supplement: Supplementary file 2 — Diagram of bird body sampling locations and burrow soil sampling depths. (DOCX 136 kb) [file 40168_2017_365_MOESM2_ESM.docx]

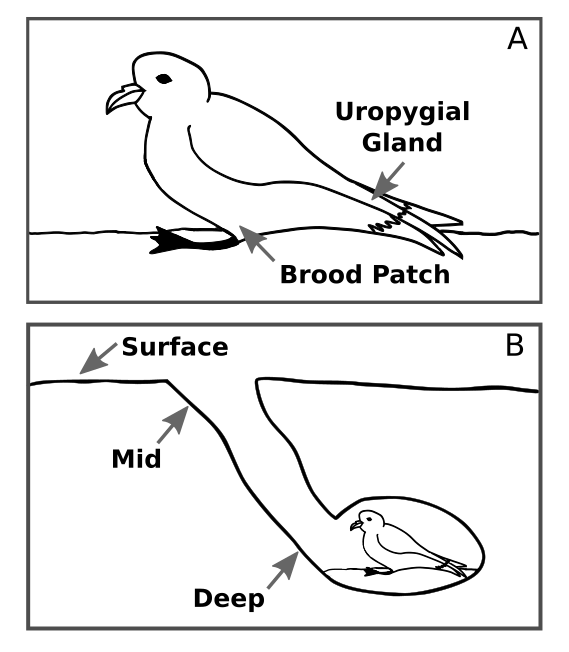


**Figure S2**- Diagrams depicting bird body site sampling locations and burrow soil sampling depths. A) Swab samples were obtained from each Leach’s storm-petrel at the uropygial gland, found on the dorsal side of the bird near the tail, and the brood patch, found on the ventral side. B) Soil samples were collected deep within the burrow, from the entrance of the burrow, and 30 cm away from the burrow entrance, referred to as deep, mid, and surface burrow soil, respectively.
