# Supplementary material for: Morphological and genetic factors shape the microbiome of a seabird species (Oceanodroma leucorhoa) more than environmental and social factors
Source: Microbiome. 2017 Oct 30;5:146. doi: 10.1186/s40168-017-0365-4 (PMC5663041; doi:10.1186/s40168-017-0365-4)
Supplement: Supplementary file 3 — Sampling summary. Two swab samples (uropygial gland and brood patch) were collected from each of 22 birds. Genotyping determined that 14 birds were female and 8 were male, and 5 male/female dyads were mated pairs. Burrow soil was sampled at 3 depths per burrow. (DOCX 20 kb) [file 40168_2017_365_MOESM3_ESM.docx]

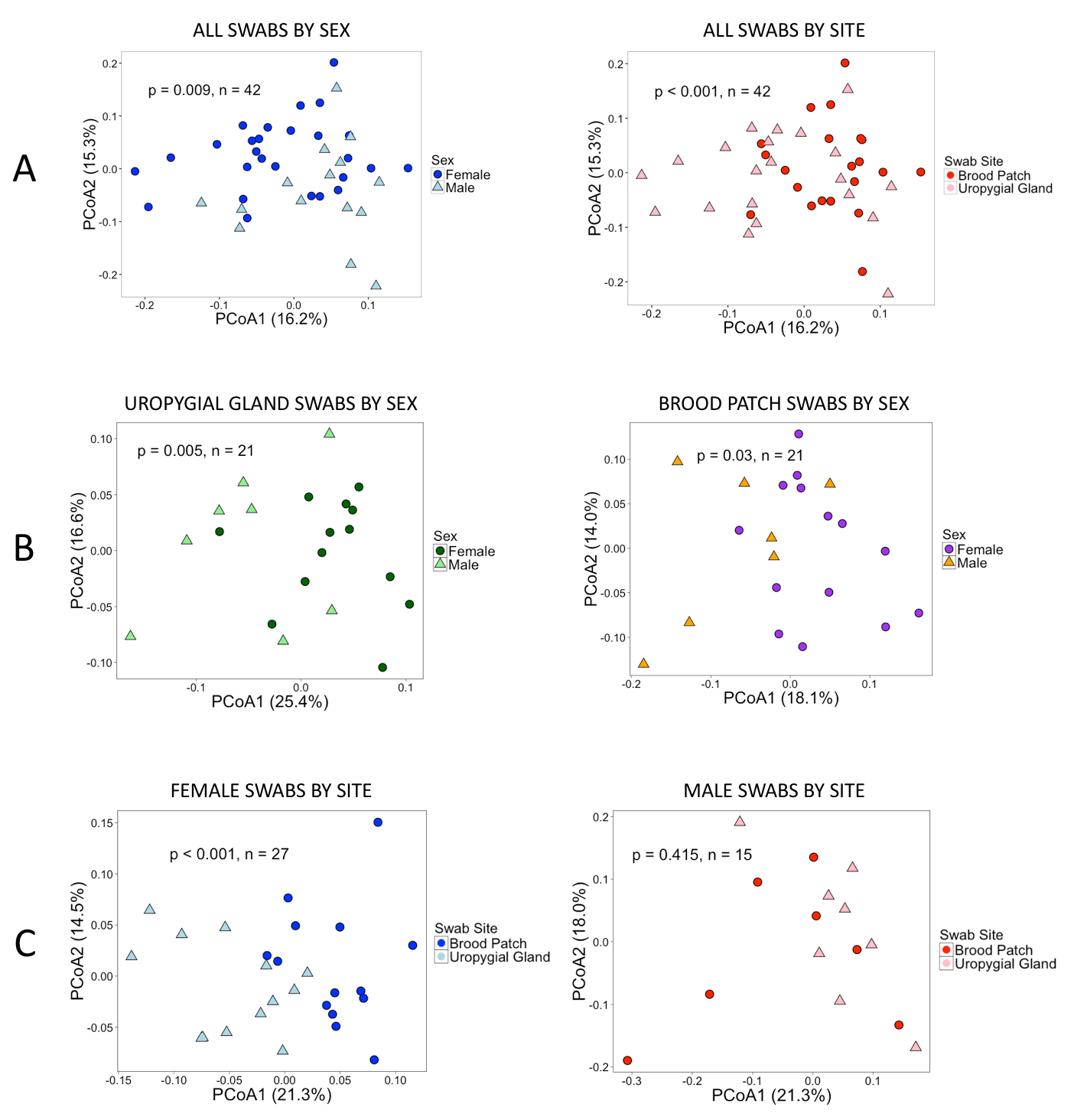


**Figure S3**- Principal coordinates of analysis of bird-associated bacterial community structure based on weighted UniFrac distances and 16S rRNA amplicons. PCoA1 and PCoA2 axes explained the highest percentage of variation in bacterial community structure, and percentages are marked on each axis. Permanova results for each comparison are indicated on each graph. Panel A indicates that bacterial communities varied by both body site and sex of the bird. Panel B indicates that the sex of the bird had a strong influence on bacterial community structure at the uropygial gland (left) and brood patch (right). Panel C shows that female birds carried different microbial communities at each of the two body sites examined (left), but body sites in male birds did not have different bacterial communities (right). Based on these results, all analyses were conducted categorically to avoid confounding results. Categories for analyses are: female brood patch swabs, female uropygial gland swabs, male brood patch swabs, and male uropygial gland swabs.
