# Supplementary material for: Morphological and genetic factors shape the microbiome of a seabird species (Oceanodroma leucorhoa) more than environmental and social factors
Source: Microbiome. 2017 Oct 30;5:146. doi: 10.1186/s40168-017-0365-4 (PMC5663041; doi:10.1186/s40168-017-0365-4)
Supplement: Supplementary file 4 — Summary of statistical analyses. Statistical analyses were performed using R, and each command and R package used is specified for each analysis. (DOCX 106 kb) [file 40168_2017_365_MOESM4_ESM.docx]

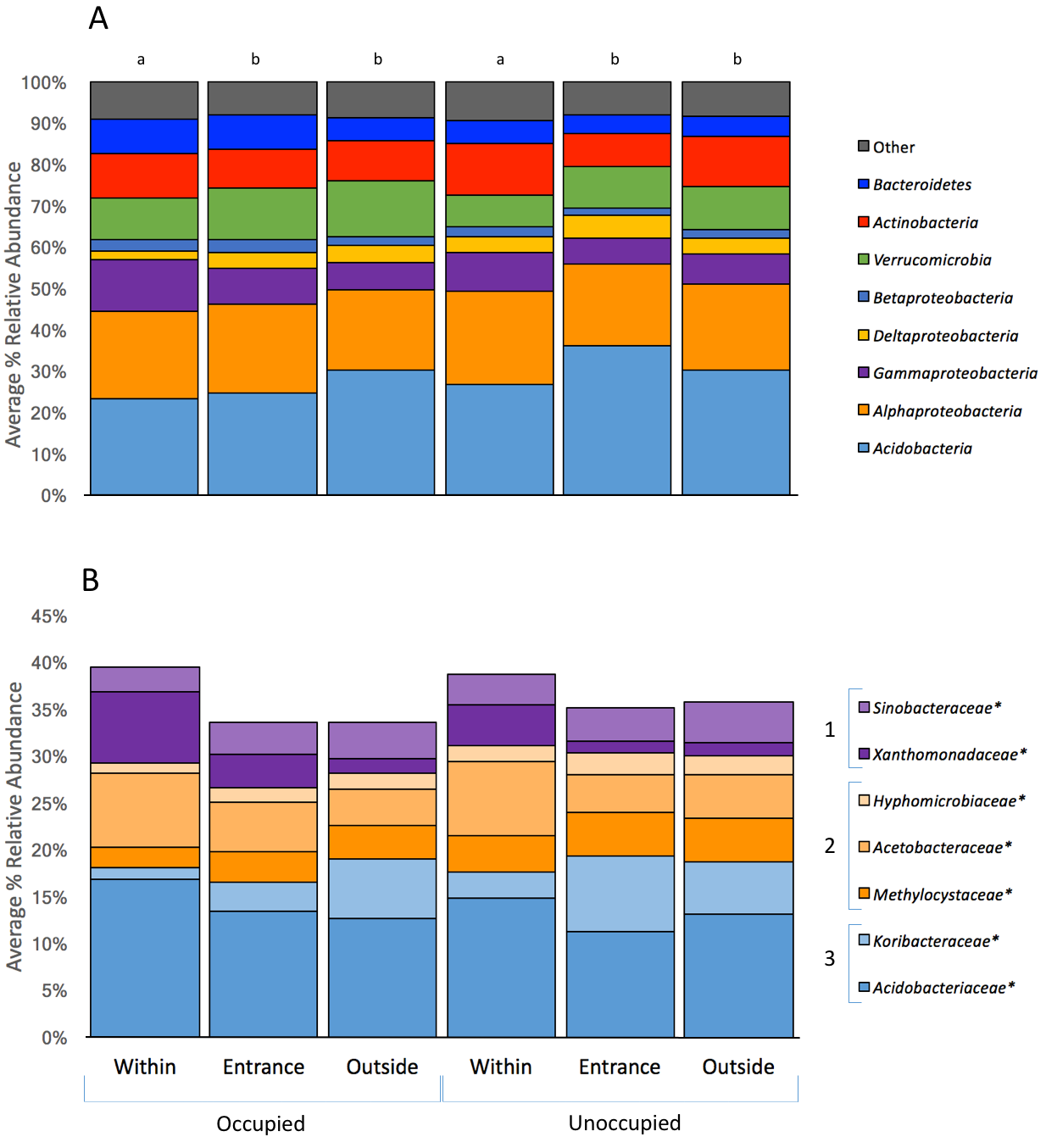


**Figure S4-** Relative abundance of top 25 bacterial species ranked by phylum (A) and family (B) among occupied and unoccupied deep, mid, and surface burrow soil categories. Burrow occupancy had no effect on bacterial community composition or structure, but burrow communities were significantly different based on depth (pseudo-F:2.796, p < 0.001, n = 78). Families that differed significantly among deep, mid, and surface burrow soil are marked with an asterisk (*). Phyla or class levels in (B) are represented as 1) Gammaproteobacteria, 2) Alphaproteobacteria, and 3) Acidobacteria.
