# Supplementary material for: Morphological and genetic factors shape the microbiome of a seabird species (Oceanodroma leucorhoa) more than environmental and social factors
Source: Microbiome. 2017 Oct 30;5:146. doi: 10.1186/s40168-017-0365-4 (PMC5663041; doi:10.1186/s40168-017-0365-4)
Supplement: Supplementary file 5 — Principal coordinates of analysis of bird-associated bacterial community structure. Bacterial communities varied by both body site and sex of the bird. The sex of the bird had a strong influence on bacterial community structure at the uropygial gland and brood patch. Female birds carried different microbial communities at each of the two body sites examined, but body sites in male birds did not have different bacterial communities. Based on these results, all analyses were conducted categorically to avoid confounding results. (DOCX 320 kb) [file 40168_2017_365_MOESM5_ESM.docx]

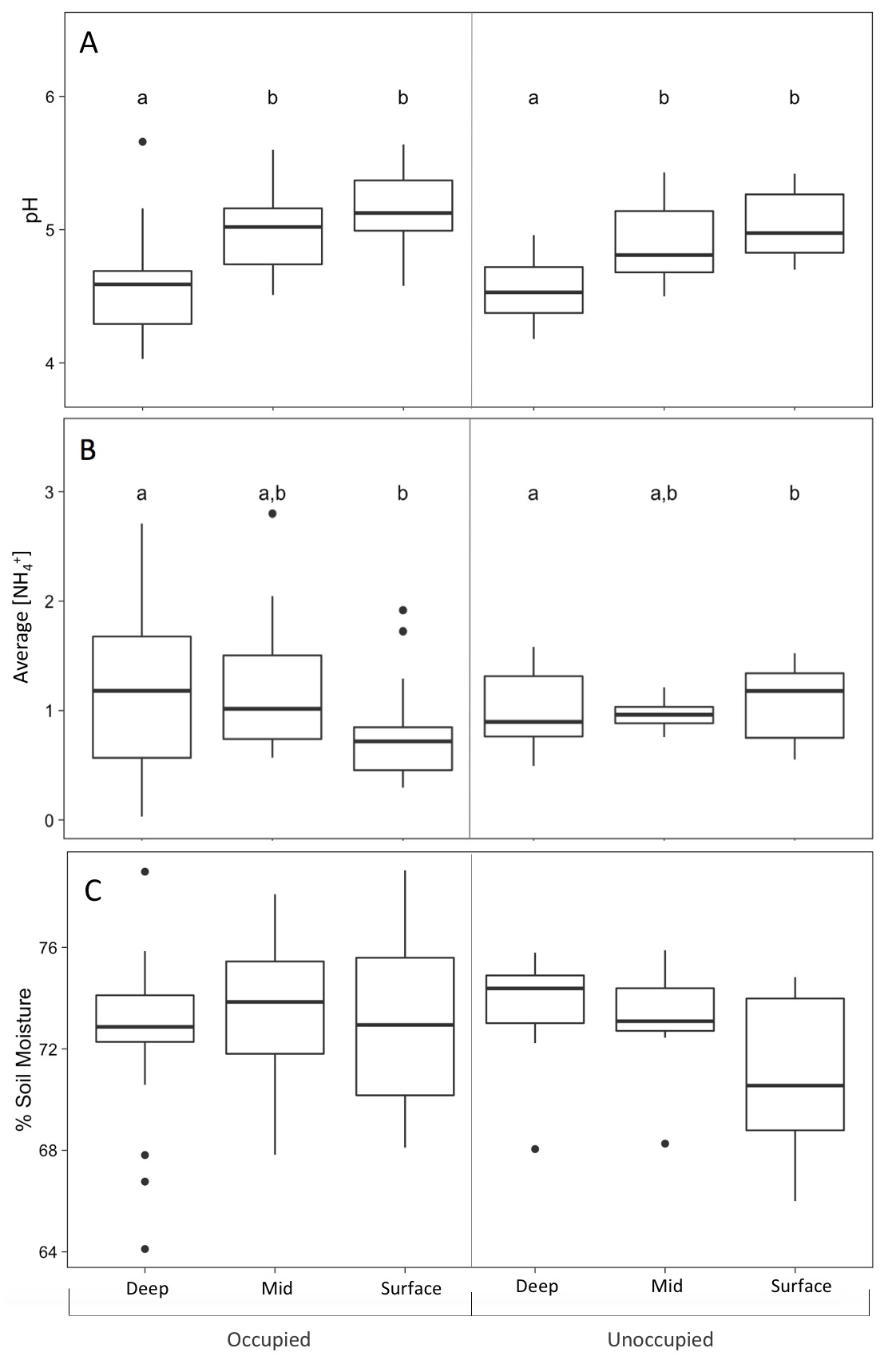


**Figure S5**- Soil pH (A), NH_4_^+^ (ppm) (B), and moisture (C) between 18 occupied and 7 unoccupied burrows at deep, mid, and surface burrow soil represented by bar and whisker plots. Boxes represent upper and lower quartiles, whiskers depict maximum and minimum values, and points are outliers. Horizontal bars within each box represent the median. Soil pH was significantly lower in deep burrow soil (F= 19.120, p < 0.001). NH_4_^+^ concentration was significantly higher in deep burrow soil compared to surface burrow soil ($\chi$^2^= 7.861, p = 0.02). Soil moisture was similar between occupied and unoccupied burrows, and was similar at all soil depths (F = 0.332, p > 0.05). Burrow occupancy had no effect on soil pH (F = 0.008, p = 0.929)), NH_4_^+^ (F = 0.543, p = 0.469), or soil moisture (F =0.377, p = 0.541).
