# Supplementary material for: Morphological and genetic factors shape the microbiome of a seabird species (Oceanodroma leucorhoa) more than environmental and social factors
Source: Microbiome. 2017 Oct 30;5:146. doi: 10.1186/s40168-017-0365-4 (PMC5663041; doi:10.1186/s40168-017-0365-4)
Supplement: Supplementary file 6 — Relative abundance of top 25 bacterial species ranked by phylum and family among occupied and unoccupied deep, mid, and surface burrow soil categories. Burrow occupancy had no effect on bacterial community composition or structure, but burrow communities were significantly different based on depth. (DOCX 293 kb) [file 40168_2017_365_MOESM6_ESM.docx]

Table S1- **Swab and soil sampling summary**. A) Two swab samples (uropygial gland and brood patch) were collected from each of 22 birds. Genotyping determined that 14 birds were female, 8 were male, and 5 male/female dyads were mated pairs. B) Sequencing revealed MHC zygosity of individual birds. C) Burrow soil was sampled at 3 depths per burrow.

1. **Bird Swab Sampling Summary**

| Skin Site | Females Sampled In Field | Female Swab Samples Remaining After Sequence Processing | Males Sampled In Field | Male Swab Samples Remaining After Sequence Processing |
| --- | --- | --- | --- | --- |
| Uropygial Gland | 14 | 13 | 8 | 8 |
| Brood Patch | 14 | 14 | 8 | 7 |
| Total Swab Samples | **28** | **27** | **16** | **15** |

1. **MHC Zygosity Summary**

| Skin Site | DAB2 Homozygous | DAB2 Heterozygous | DAB2 Genotype Unknown |
| --- | --- | --- | --- |
| Uropygial Gland, Female | 4 | 8 | 1 |
| Brood Patch, Female | 5 | 8 | 1 |
| Uropygial Gland, Male | 3 | 5 | 0 |
| Brood Patch, Male | 2 | 5 | 0 |

1. **Soil Sampling Summary**

| Soil Sample Type | Occupied Burrows | Burrows Occupied By Paired Birds | Unoccupied Burrows | Total |
| --- | --- | --- | --- | --- |
| Deep Burrow Soil | 18 | 5 | 7 | 25 |
| Mid Burrow Soil | 18 | 5 | 7 | 25 |
| Surface Burrow Soil | 18 | 5 | 7 | 25 |
| Total Soil Samples | **54** | **15** | **21** | **75** |
