# Supplementary material for: Morphological and genetic factors shape the microbiome of a seabird species (Oceanodroma leucorhoa) more than environmental and social factors
Source: Microbiome. 2017 Oct 30;5:146. doi: 10.1186/s40168-017-0365-4 (PMC5663041; doi:10.1186/s40168-017-0365-4)
Supplement: Supplementary file 7 — Soil abiotic properties between 18 occupied and 7 unoccupied burrows at deep, mid, and surface burrow soil. Soil pH was significantly lower in deep burrow soil, and NH4 + concentration was significantly higher in deep burrow soil compared to surface burrow soil. Soil moisture was similar between occupied and unoccupied burrows and was similar at all soil depths, while burrow occupancy had no effect on soil pH or soil moisture. (DOCX 153 kb) [file 40168_2017_365_MOESM7_ESM.docx]

Table S2- **Summary of statistical analyses performed.** F = female, M = male, U = uropygial gland, B = brood patch. Statistical analyses were performed using R, and each command and R package used is specified for each analysis.

| Category | Measurement | Samples or Groups | Test | R Command(s) | R Package(s) |
| --- | --- | --- | --- | --- | --- |
| Individual Effects | Community richness | Within-sample | Shannon-Weaver | diversity | vegan |
|  | Differences in Shannon diversity | FvM, U; FvM, B;  F, UvB; M, UvB | Wilcoxon rank sum | wilcox.test | stats |
|  | Between-sample beta diversity | FvM, U; FvM, B;  F, UvB; M, UvB | Weighted UniFrac, PERMANOVA | distance, adonis | phyloseq, vegan |
|  | Similarity percentage | FvM, U; FvM, B;  F, UvB; M, UvB | Simper | simper | vegan |
|  | Morphological and genetic overlays | FvM, U; FvM, B;  F, UvB; M, UvB | Vector overlay | envfit | vegan |
|  | Community relative abundances | FvM, U; FvM, B;  F, UvB; M, UvB | Power transformation, one-way ANOVA | powerTransform, anova, testInteractions | car  stats  phia |
| Environmental Effects | Abiotic soil properties (NH4+, soil moisture, pH) | Deep, mid, surface soils from occupied and unoccupied burrows | Two-way ANOVA, Kruskal-Wallis | anova, kruskal.test | car,  stats |
|  | Correlation between UniFrac and physical distance matrices | Bird community distance matrices vs. physical burrow distance matrix; burrow community distance matrices vs. physical burrow distance matrix | Calculated distance between points, Mantel test for similiarity of two matrices | spDists, mantel.test | sp, ape |
|  | Percent shared OTUs between birds and burrows | F vs. Burrow, U; F vs. Burrow, B; Male vs. Burrow, U; Male vs. Burrow, B. | One-way ANOVA | anova | car |
| Social Effects | Percent shared OTUs between birds | Female vs. mate or randomly-assigned non-mate; Male vs. mate or randomly-assigned non-mate | Welch’s two-sample t-test | t.test | stats |
