# Supplementary material for: Morphological and genetic factors shape the microbiome of a seabird species (Oceanodroma leucorhoa) more than environmental and social factors
Source: Microbiome. 2017 Oct 30;5:146. doi: 10.1186/s40168-017-0365-4 (PMC5663041; doi:10.1186/s40168-017-0365-4)
Supplement: Supplementary file 8 — Comparison of burrow soil distance matrices with geographical distance. Weighted and unweighted UniFrac discance matrices were compared to geographical burrow distance. At all burrow soil depths, community presence/absence significantly correlated with geographical burrow distance and bacterial community structure from mid and surface burrow soil significantly correlated with geographical burrow distance. (DOCX 17 kb) [file 40168_2017_365_MOESM8_ESM.docx]

Table S3- **Comparison of burrow soil distance matrices with geographical distance**. Weighted and unweighted UniFrac discance matrices were compared to geographical burrow distance using the Mantel test. Unweighted UniFrac measures community presence/absence while weighted UniFrac measures community structure. At all burrow soil depths, community presence/absence significantly correlated with geographical burrow distance, and bacterial community structure from mid and surface burrow soil significantly correlated with geographical burrow distance.

| Comparison | Distance Matrix | Test Statistic R | P Value | n |
| --- | --- | --- | --- | --- |
| Deep burrow soil, burrow Distance | Weighted Unifrac  Unweighted Unifrac | 0.072  0.118 | 0.168  **0.053** | 25  25 |
| Mid burrow soil, burrow Distance | Weighted Unifrac  Unweighted Unifrac | 0.141  0.240 | **0.043**  **0.003** | 25  25 |
| Surface burrow soil, burrow Distance | Weighted Unifrac  Unweighted Unifrac | 0.190  0.231 | **0.014**  **0.004** | 25  25 |
