# Supplementary material for: Morphological and genetic factors shape the microbiome of a seabird species (Oceanodroma leucorhoa) more than environmental and social factors
Source: Microbiome. 2017 Oct 30;5:146. doi: 10.1186/s40168-017-0365-4 (PMC5663041; doi:10.1186/s40168-017-0365-4)
Supplement: Supplementary file 9 — Families represented within top phyla. In Fig. 1b, families representing the top 20 most abundant species are shown. The remaining families within each phylum are shown in Fig. 1 are listed in this table. (DOCX 121 kb) [file 40168_2017_365_MOESM9_ESM.docx]

*Table S4*- **Families represented within top phyla**. In figure 1B, families representing the top 20 most abundant OTUs are represented. The remaining families that did not represent the top 20 most abundant OTUs are represented below. Average % relative abundance representing all sexes and body sites is shown in parentheses after each family name.

| \| ***Firmicutes*** \| \| --- \| \| *Listeriaceae (4.89)* \| \| *Paenibacillaceae (1.04)* \| \| *Bacillaceae (1.00)* \| \| *[Tissierellaceae] (0.44)* \| \| *Clostridiaceae (0.42)* \| \| *Enterococcaceae (0.36)* \| \| *Streptococcaceae (0.31)* \| \| *Aerococcaceae (0.25)* \| \| *Leuconostocaceae (0.20)* \| \| *[Exiguobacteraceae] (0.17)* \| \| *Christensenellaceae (0.17)* \| \| *Lactobacillaceae (0.17)* \| \| *Staphylococcaceae (0.13)* \| \| *Ruminococcaceae (0.08)* \| \| *Erysipelotrichaceae (0.04)* \| \| *Planococcaceae (0.04)* \| \| *Alicyclobacillaceae (0.03)* \| \| *Lachnospiraceae (0.01)* \| \| *Syntrophomonadaceae (<0.01)* \| | \| ***Bacteroidetes*** \| \| --- \| \| *[Weeksellaceae] (11.97)* \| \| *Chitinophagaceae (4.46)* \| \| *Cytophagaceae (3.28)* \| \| *Flavobacteriaceae (1.91)* \| \| *Porphyromonadaceae (0.44)* \| \| *Saprospiraceae (0.41)* \| \| *[Paraprevotellaceae] (0.24)* \| \| *Prevotellaceae (0.08)* \| \| *Cryomorphaceae (0.03)* \| \| *[Amoebophilaceae] (<0.01)* \| \|  \| \| ***Betaproteobacteria*** \| \| *Comamonadaceae (5.51)* \| \| *Alcaligenaceae (0.71)* \| \| *Burkholderiaceae (0.55)* \| \| *Procabacteriaceae (0.37)* \| \| *Rhodocyclaceae (0.33)* \| \| *Methylophilaceae (0.22)* \| \| *EB1003 (0.05)* \| \| *Nitrosomonadaceae (<0.01)* \| | \| ***Actinobacteria*** \| \| --- \| \| *Actinomycetaceae (4.51)* \| \| *Microbacteriaceae (4.44)* \| \| *Conexibacteraceae (4.36)* \| \| *Dermacoccaceae (3.85)* \| \| *Frankiaceae (3.03)* \| \| *Nocardiaceae (2.94)* \| \| *Streptomycetaceae (1.09)* \| \| *Patulibacteraceae (0.84)* \| \| *Brevibacteriaceae (0.63)* \| \| *Sporichthyaceae (0.52)* \| \| *Nocardioidaceae (0.51)* \| \| *Pseudonocardiaceae (0.36)* \| \| *Actinospicaceae (0.35)* \| \| *Nakamurellaceae (0.35)* \| \| *Coriobacteriaceae (0.31)* \| \| *Mycobacteriaceae (0.29)* \| \| *Micrococcaceae (0.25)* \| \| *Kineosporiaceae (0.25)* \| \| *Dermabacteraceae (0.07)* \| | \| ***Actinobacteria cont’d...*** \| \| --- \| \| *Cellulomonadaceae (0.06)* \| \| *EB1017 (0.05)* \| \| *Gaiellaceae (0.04)* \| \| *Micromonosporaceae (0.04)* \| \| *Intrasporangiaceae (0.04)* \| \| *Geodermatophilaceae (0.03)* \| \| *Gordoniaceae (0.03)* \| \| *Euzebyaceae (0.03)* \| \| *C111 (0.02)* \| \| *Dietziaceae (0.02)* \| \| *Williamsiaceae (0.01)* \| \| *AK1AB1_02E (0.01)* \| \| *Rubrobacteraceae (0.01)* \| \| *Solirubrobacteraceae (<0.01)* \| \| *ACK-M1 (<0.01)* \| \|  \| | \| ***Alphaproteobacteria*** \| \| --- \| \| *Acetobacteraceae (16.89)* \| \| *Methylobacteriaceae (14.08)* \| \| *Methylocystaceae (8.94)* \| \| *Caulobacteraceae (7.35)* \| \| *Hyphomicrobiaceae (3.30)* \| \| *Bradyrhizobiaceae (2.44)* \| \| *Rhizobiaceae (1.93)* \| \| *Bartonellaceae (1.76)* \| \| *Rhodospirillaceae (1.52)* \| \| *Rhodobacteraceae (1.13)* \| \| *Beijerinckiaceae (1.02)* \| \| *Brucellaceae (0.75)* \| \| *Phyllobacteriaceae (0.52)* \| \| *Rickettsiaceae (0.22)* \| \| *Xanthobacteraceae (0.21)* \| \| *Aurantimonadaceae (0.18)* \| \| *Erythrobacteraceae (0.15)* \| \| *Hyphomonadaceae (0.04)* \| | \| ***Gammaproteobacteria*** \| \| --- \| \| *Enterobacteriaceae (9.02)* \| \| *Coxiellaceae (5.13)* \| \| *Cardiobacteriaceae (3.45)* \| \| *Sinobacteraceae (3.11)* \| \| *Alteromonadaceae (0.60)* \| \| *Pasteurellaceae (0.51)* \| \| *Legionellaceae (0.44)* \| \| *Aeromonadaceae (0.23)* \| \| *Vibrionaceae (0.21)* \| \| *[Chromatiaceae] (0.03)* \| \| *Salinisphaeraceae (0.02)* \| \|  \| |
| --- | --- | --- | --- | --- | --- | --- | --- | --- | --- | --- | --- | --- | --- | --- | --- | --- | --- | --- | --- | --- | --- | --- | --- | --- | --- | --- | --- | --- | --- | --- | --- | --- | --- | --- | --- | --- | --- | --- | --- | --- | --- | --- | --- | --- | --- | --- | --- | --- | --- | --- | --- | --- | --- | --- | --- | --- | --- | --- | --- | --- | --- | --- | --- | --- | --- | --- | --- | --- | --- | --- | --- | --- | --- | --- | --- | --- | --- | --- | --- | --- | --- | --- | --- | --- | --- | --- | --- | --- | --- | --- | --- | --- | --- | --- | --- | --- | --- | --- | --- | --- | --- | --- | --- | --- | --- | --- | --- | --- | --- | --- | --- | --- | --- | --- | --- |
